# Focus on proteolysis

**DOI:** 10.1093/plcell/koae182

**Published:** 2024-06-25

**Authors:** Nancy A Eckardt, Pascal Genschik, Liwen Jiang, Xin Li, Marisa S Otegui, Ari Sadanandom, Steven H Spoel, Klaas J van Wijk, Dolf Weijers

**Affiliations:** The Plant Cell, American Society of Plant Biologists, USA; The Plant Cell, American Society of Plant Biologists, USA; Institut de Biologie Moléculaire des Plantes, CNRS, Université de Strasbourg, 12, rue du Général Zimmer, Strasbourg 67084, France; The Plant Cell, American Society of Plant Biologists, USA; School of Life Sciences, Centre for Cell and Developmental Biology, State Key Laboratory of Agrobiotechnology, The Chinese University of Hong Kong, Shatin, New Territories, Hong Kong, China; The Plant Cell, American Society of Plant Biologists, USA; Department of Botany and Michael Smith Laboratories, University of British Columbia, Vancouver, BC, Canada V6T 1Z4; The Plant Cell, American Society of Plant Biologists, USA; Department of Botany and Center for Quantitative Cell Imaging, University of Wisconsin-Madison, Madison, WI 53706, USA; The Plant Cell, American Society of Plant Biologists, USA; Department of Biosciences, Durham University, Durham DH1 3LE, UK; The Plant Cell, American Society of Plant Biologists, USA; Institute of Molecular Plant Sciences, School of Biological Sciences, University of Edinburgh, The King’s Buildings, Edinburgh EH9 3BF, UK; The Plant Cell, American Society of Plant Biologists, USA; Section of Plant Biology, School of Integrative Plant Sciences (SIPS), Cornell University, Ithaca, NY 14853, USA; The Plant Cell, American Society of Plant Biologists, USA; Laboratory of Biochemistry, Wageningen University, Wageningen 6708WE, The Netherlands

Proteolysis is an essential cellular function mediating the processing and turnover of proteins to remove damaged or inactive proteins, alter protein function (binding or enzymatic activities), and ensure appropriate protein stoichiometries in the cell. The post-translational control of protein stability is also a central feature of cellular signaling in eukaryotes, i.e. regulating the turnover of important regulatory proteins, and is crucial for almost all aspects of plant biology, including vegetative growth, development, reproduction, and stress responses. Proteolysis plays a central role in hormone signaling pathways, plant defense against pests and pathogens, abiotic stress responses, and basic cell functions like the cell cycle, metabolism, organellar biogenesis and maintenance, and senescence. Compared with animals, plant genomes encode a highly expanded number of components related to proteolytic pathways including the ubiquitin-proteasome system, autophagy, programmed cell death, endosomal trafficking, and organelle-associated protein degradation. Recent advances in molecular genetics and cell biology, microscopy and high-resolution imaging, in vivo labeling, proteomics, mass spectrometry, and structural biology have led to new insights and understanding in many areas of plant proteolysis, including autophagy of chloroplasts and other organelles, degradation of membrane proteins, the discovery of plant N-degron pathways, and proteolytic processing involved in plant development and environmental responses (immunity and abiotic stress). Knowledge of plant proteolytic systems is also important for agriculture and plant breeding, given the impact on plant growth and development related to yield, as well as plant resistance to pests and diseases.

This focus issue on plant proteolysis includes 2 letters to the editor, 1 commentary, 9 review articles, and 8 original research articles. The 2 letters address the question of whether the polyubiquitin pathway operates inside intact chloroplasts. The question is posed by van Wijk and Adam (2024), with a Reply from Jarvis et al. (2024). Researchers on both sides of the debate make valid points to be considered. We eagerly await more definitive data to arrive at a complete understanding of protein degradation inside chloroplasts. The commentary by Eckardt et al. (2024) includes expert opinions on compelling open questions in plant proteolysis research. De Roij et al. (2024) summarize the discovery of the auxin receptor and a core auxin signaling hub that relies on the degradation of Aux/IAA transcriptional inhibitors, highlighting the interconnection of proteolytic systems in auxin signaling. Genschik et al. (2024) review evidence that key components of the RNA silencing machinery in plants are regulated by proteolysis during plant development and by microbial hijacking of endogenous proteases. Liu et al. (2024) summarize the roles various proteases play in plant immunity, highlighting studies focused on engineering components of proteolysis to achieve broad-spectrum resistance without yield reduction in crop species. Three reviews cover proteolysis in relation to organelles. Otegui et al. (2024) review mechanisms that control the vacuolar degradation of plant organelles, emphasizing autophagy and crosstalk with other pathways. Zhuang et al. (2024) review the biogenesis of the autophagosome in plants, the double-membrane structure that delivers cargo to the vacuole during autophagy, with emphasis on autophagosome-organelle interactions under abiotic stress conditions. Van Wijk (2024) then reviews the proteolysis network within chloroplasts and non-photosynthetic plastids that is distributed across the intra-chloroplast compartments of lumen, thylakoid, stroma, and plastid envelopes. Three more reviews focus on different aspects of how proteins are recognized and tagged for proteolysis. Isono et al. (2024) review the complex process of how specific proteins are targeted for various degradation pathways in plants. Vogel and Isono (2024) review the process and components of deubiquitylation in plants and its importance in understanding the ubiquitin code that targets proteins for degradation by the ubiquitin-proteasome system and other outcomes. Finally, Ghosh et al. (2024) review the evolution of the small ubiquitin-like modifier (SUMO) system in plants with an emphasis on the relation to stress adaptation.

In the first of 8 original research articles, Huang and Rojas-Pierce (2024) present a breakthrough report in which they describe an inducible protein degradation system in plants that might be used to study the loss of any cytoplasmic plant protein with high-temporal resolution. Kourelis et al. (2024) report on the bioengineering of secreted proteases from eggplant, tobacco, and tomato, triggering Avr2/Cf-2-dependent immunity to the fungal pathogen *Cladosporium fulvum*. Wu et al. (2024) study the phenomenon of virus-induced drought tolerance in common bean and show that a viral small interfering RNA-host plant mRNA pathway modulates virus-induced drought tolerance by enhancing autophagy. Feiz et al. (2024) find that the jasmonic acid COI1 F-box receptor proteins regulate DELLA protein levels, by triggering proteasome-dependent DELLA degradation, to regulate growth, photosynthetic efficiency, and defense pathways in maize. Finally, 4 articles report on the function of different E3 ubiquitin ligases in plant development and stress responses. Zhang et al. (2024) studied the large plant protein BIG, a homolog of an E3 ubiquitin ligase in mammals, whose function and molecular activity in plants is unclear. They report the interaction of BIG with other E3 ligase components that interact with the proteasome, and a potential function in suberin deposition and plant response to hypoxia. Yue et al. (2024) investigated the protein LARGE2 in rice, the mutant of which produces large panicles with increased grain size, and report that this HECT-domain E3 ubiquitin ligase functions together with other E3 ligase components to control rice panicle and grain size. Yu et al. (2024) show that 2 E3 ubiquitin ligases, MAC3A and MAC3B, mediate degradation of the transcription factor ERF13, thereby promoting lateral root emergence in Arabidopsis. Du et al. (2024) report that the ABA-responsive E3 ligase PUB35 forms a module with ABI5 BINDING PROTEIN1 (AFP1), which negatively regulates ABA signaling by mediating the ubiquitylation and degradation of the transcription factor ABI5.

These articles contribute a wealth of new information and insightful review and discussion of the state of the art of proteolysis research in plants. We hope that this focus issue stimulates further progress in this arena. We encourage authors to continue to submit their best work on plant proteolysis to *The Plant Cell*. Articles published in this area within 8 to 12 mo of this focus issue will be added to an online collection on proteolysis, building on the articles presented here.

## References

[koae182-B1] de Roij M , Borst JW, Weijers D. Protein degradation in auxin response. Plant Cell. 2024:36(9):3025–3035. 10.1093/plcell/koae125 PMC11371164 38652687

[koae182-B2] Du C , Liu M, Yan Y, Guo X, Cao X, Jiao Y, Zheng J, Ma Y, Xie Y, Li H, et al The U-Box E3 ubiquitin ligase PUB35 negatively regulates ABA signaling through AFP1-mediated degradation of ABI5. Plant Cell. 2024:36(9):3277–3297. 10.1093/plcell/koae194 PMC11371175 38924024

[koae182-B3] Eckardt NA , Avin-Wittenberg T, Bassham DC, Chen P, Chen Q, Fang J, Genschik P, Ghifari AS, Guercio AM, Gibbs DJ, et al The lowdown on breakdown: open questions in plant proteolysis. Plant Cell. 2024:36(5):1183–1185. 10.1093/plcell/koae076 38980154 PMC11371169

[koae182-B4] Feiz L , Shyu C, Wu S, Ahern KR, Gull I, Rong Y, Artymowicz CJ, Piñeros MA, Fei Z, Brutnell TP, et al COI1 F-box proteins regulate DELLA protein levels, growth, and photosynthetic efficiency in maize. Plant Cell. 2024:36(9):3237–3259. 10.1093/plcell/koae161 PMC11371192 38801745

[koae182-B5] Genschik P , Schiaffini M, Lechner E. Proteolytic control of the RNA silencing machinery. Plant Cell. 2024:36(9):2997–3008. 10.1093/plcell/koae075 PMC11371168 38456220

[koae182-B6] Ghosh S , Mellado Sánchez M, Sue-Ob K, Roy D, Jones A, Blazquez MA, Sadanandom A. Charting the evolutionary path of the SUMO modification system in plants reveals molecular hardwiring of development to stress adaptation. Plant Cell. 2024:36(9):3131–3144. 10.1093/plcell/koae192 PMC11371177 38923935

[koae182-B7] Huang L , Rojas-Pierce M. Rapid depletion of target proteins in plants by an inducible protein degradation system. Plant Cell. 2024:36(9):3145–3161. 10.1093/plcell/koae072 PMC11371150 38446628

[koae182-B8] Isono E , Li J, Pulido P, Siao W, Spoel SH, Wang Z, Zhuang X, Trujillo M. Protein degrons and degradation: exploring substrate recognition and pathway selection in plants. Plant Cell. 2024:36(9):3074–3098. 10.1093/plcell/koae141 PMC11371205 38701343

[koae182-B9] Jarvis RP , LiJ, LinR, LingQ, LyuY, SunY, YaoZ. Reply: does the polyubiquitination pathway operate inside intact chloroplasts to remove proteins?Plant Cell. 2024:36(9):2990–2996. 10.1093/plcell/koae10538738499

[koae182-B10] Kourelis J , Schuster M, Demir F, Mattinson O, Krauter S, Kahlon P, O'Grady R, Royston S, Bravo-Cazar AL, Mooney BC, et al Bioengineering secreted proteases converts divergent Rcr3 orthologs and paralogs into extracellular immune co-receptors. Plant Cell. 2024:36(9):3260–3276. 10.1093/plcell/koae183 PMC11371160 38923940

[koae182-B11] Liu Y , Jackson E, Liu X, Huang X, van der Hoorn RAL, Zhang Y, Li X. Proteolysis in plant immunity. Plant Cell. 2024:36(9):3099–3115. 10.1093/plcell/koae142 PMC11371161 38723588

[koae182-B12] Otegui MS , Steelheart C, Ma W, Ma J, Kang B-H, Sanchez De Medina Hernandez V, Dagdas Y, Gao C, Goto-Yamada S, Oikawa K, et al Vacuolar degradation of plant organelles. Plant Cell. 2024:36(9):3036–3056. 10.1093/plcell/koae128 PMC11371181 38657116

[koae182-B13] van Wijk KJ . Intra-chloroplast proteases: A holistic network view of chloroplast proteolysis. Plant Cell. 2024:36(9):3116–3130. 10.1093/plcell/koae178 PMC11371162 38884601

[koae182-B14] van Wijk KJ , AdamZ. Does the polyubiquitination pathway operate inside intact chloroplasts to remove proteins?Plant Cell. 2024:36(9):2984–2989. 10.1093/plcell/koae10438683741

[koae182-B15] Vogel K , Isono E. Erasing marks: functions of plant deubiquitylating enzymes in modulating the ubiquitin code. Plant Cell. 2024:36(9):3057–3073. 10.1093/plcell/koae129 PMC11371157 38656977

[koae182-B16] Wu X , Chen S, Zhang Z, Zhou W, Sun T, Ning K, Xu M, Ke X, Xu P. A viral small interfering RNA-host plant mRNA pathway modulates virus-induced drought tolerance by enhancing autophagy. Plant Cell. 2024:36(9):3219–3236. 10.1093/plcell/koae158 PMC11371139 38801738

[koae182-B17] Yu Z , Qu X, Lv B, Li X, Sui J, Yu Q, Ding Z. MAC3A and MAC3B mediate degradation of the transcription factor ERF13 and thus promote lateral root emergence. Plant Cell. 2024:36(9):3162–3176. 10.1093/plcell/koae047 PMC11371146 38366565

[koae182-B18] Yue Z , Wang Z, Yao Y, Liang Y, Li J, Yin K, Li R, Li Y, Ouyang Y, Xiong L, et al Variation in WIDTH OF LEAF AND GRAIN contributes to grain and leaf size by controlling LARGE2 stability in rice. Plant Cell. 2024:36(9):3201–3218. 10.1093/plcell/koae136 PMC11371194 38701330

[koae182-B19] Zhang H , Rundle C, Winter N, Miricescu A, Mooney BC, Bachmair A, Graciet E, Theodoulou FL. BIG enhances Arg/N-degron pathway-mediated protein degradation to regulate Arabidopsis hypoxia responses and suberin deposition. Plant Cell. 2024:36(9):3177–3200. 10.1093/plcell/koae117 PMC11371152 38608155

[koae182-B20] Zhuang X , Li B, Jiang L. Autophagosome biogenesis and organelle homeostasis in plant cells. Plant Cell. 2024:36(9):3009–3024. 10.1093/plcell/koae099 PMC11371174 38536783

